# Evolution Mechanism of Volume Parameters and Gradation Optimization Method for Asphalt Mixtures Based on Dual-Domain Fractal Theory

**DOI:** 10.3390/ma19030488

**Published:** 2026-01-26

**Authors:** Bangyan Hu, Zhendong Qian, Fei Zhang, Yu Zhang

**Affiliations:** 1 Intelligent Transportation System Research Center, Southeast University, Nanjing 211189, China; qianzd@seu.edu.cn; 2 College of Civil Engineering and Architecture, Xinjiang University, Urumqi 830017, China; zfxj1007@126.com; 3 Nanjing Modern Multimodal Transportation Laboratory, Nanjing 211100, China; 4 Xinjiang Transportation Planning, Survey and Design Institute Co., Ltd., Urumqi 830006, China; 5 Zhejiang Province Engineering Research Center for Applied Technology of Digital Highways, Zhejiang Institute of Communications, Hangzhou 311112, China; zhangyu0614@zjvtit.edu.cn

**Keywords:** asphalt mixture, dual-domain fractal theory, volumetric parameters, gradation optimization, grey relational analysis

## Abstract

The primary objective of this study is to bridge the gap between descriptive geometry and mechanistic design by establishing a dual-domain fractal framework to analyze the internal architecture of asphalt mixtures. This research quantitatively assesses the sensitivity of volumetric indicators—namely air voids (VV), voids in mineral aggregate (VMA), and voids filled with asphalt (VFA)—by employing the coarse aggregate fractal dimension (*D_c_*), the fine aggregate fractal dimension (*D_f_*), and the coarse-to-fine ratio (k) through Grey Relational Analysis (GRA). The findings demonstrate that whereas *D_f_* and k substantially influence macro-volumetric parameters, the mesoscopic void fractal dimension (D_V_) remains structurally unchanged, indicating that gradation predominantly dictates void volume rather than geometric intricacy. Sensitivity rankings create a prevailing hierarchy: Process Control (Compaction) > Skeleton Regulation (*D_c_*) > Phase Filling (P_b_) > Gradation Adjustment (k, *D_f_*). *D_c_* is recognized as the principal regulator of VMA, while binder content (Pb) governs VFA. A “Robust Design” methodology is suggested, emphasizing *D_c_* to stabilize the mineral framework and reduce sensitivity to construction variations. A comparative investigation reveals that the optimized gradation (OG) achieves a more stable volumetric condition and enhanced mechanical performance relative to conventional empirical gradations. Specifically, the OG group demonstrated a substantial 112% enhancement in dynamic stability (2617 times/mm compared to 1230 times/mm) and a 75% increase in average film thickness (AFT), while ensuring consistent moisture and low-temperature resistance. In conclusion, this study transforms asphalt mixture design from empirical trial-and-error to a precision-engineered methodology, providing a robust instrument for optimizing the long-term durability of pavements in extreme cold and arid environments.

## 1. Introduction

The longevity and structural soundness of asphalt pavements are mostly determined by the internal composition of the asphalt mixture [1]. The performance of this multi-phase composite material, including its rutting resistance at high temperatures and fatigue endurance, is predominantly influenced by the spatial configuration of the mineral aggregate skeleton and the distribution of air voids [2,3]. Modern design theories, such as Stone Matrix Asphalt (SMA) and Superpave [4], strive to create a stable “skeleton-filling” structure where coarse aggregates form a load-bearing framework and fine aggregates/mastic provide dense filling [5]. Thus, aggregate gradation serves as the principal mechanism for regulating this volumetric structure [6]. Nonetheless, conventional design procedures, such as the Bailey Method and Superpave, predominantly rely on empirical approaches and necessitate significant, time-intensive laboratory testing [7,8]. These approaches often struggle to accurately predict essential volumetric indicators, such as Air Voids (VV) and Voids in Mineral Aggregate (VMA) [9], leading to an inefficient trial-and-error design process that lacks a clear explanation of the underlying physical mechanisms of volumetric evolution [10,11,12].

The complexity of asphalt mixtures arises from the multiscale nature of aggregate particles, which exhibit significant self-similarity in their distribution. To capture this complexity, fractal geometry has emerged as a robust mathematical framework for quantifying the packing behavior of aggregates [13,14]. By employing the fractal dimension as a continuous metric, researchers can move beyond discrete empirical intervals to establish analytical links between gradation morphology and macroscopic performance [2,15,16,17]. Despite these advancements, most existing fractal models treat the gradation as a single, homogeneous domain [18]. This “single-fractal” approach often masks the distinct mechanical roles of different particle size ranges. In reality, the coarse aggregate fraction (>2.36 mm or 4.75 mm) governs the shear resistance through grain-to-grain contact, while the fine aggregate fraction acts as a semi-solid matrix [17,19]. A single fractal dimension fails to capture the transition in packing logic between these two domains, necessitating a more granular, dual-domain fractal approach to describe the mixture’s internal structure accurately [13,20,21].

Furthermore, the volumetric state of a compacted mixture is not a static geometric property determined solely by its initial gradation. It is the result of a dynamic, energy-driven process where particles undergo reorganization and orientation under external loads [22,23,24]. This evolution is governed by the interaction between the “Structural Domain” (the fractal architecture of the aggregates) and the “Process Domain” (fabrication variables such as asphalt content P_b_ and compaction energy) [25,26,27]. Asphalt binder acts as both a lubricant facilitating particle movement and a filling agent occupying the interstitial voids [28]. Current research often overlooks this coupled effect, leading to designs that may meet volumetric targets in the lab but exhibit high sensitivity to construction variations in the field [29]. There is a critical need to understand the hierarchical influence of these parameters to ensure that a gradation is not only “optimal” but also “robust” against unavoidable fluctuations in material proportions and compaction effort [30].

However, translating this need for robustness into engineering practice is fundamentally hindered by the limitations of current design paradigms. While the Superpave volumetric system [31] provides indispensable empirical benchmarks, it lacks the theoretical depth to quantify how internal geometry responds to energy-driven changes. Conversely, although Discrete Element Modeling (DEM) effectively captures individual particle behaviors at the microscopic scale, its application in large-scale pavement simulations is fundamentally constrained by a prohibitively high computational cost, particularly when accounting for complex aggregate geometries and arrangements [32].

To bridge this gap, this research proposes a ‘Dual-domain Fractal Framework’ that improves upon traditional single-fractal models by decoupling the gradation into a coarse aggregate fractal dimension (*D_c_*) and a fine aggregate fractal dimension (*D_f_*). Unlike conventional trial-and-error procedures, this approach systematically investigates the synergistic interaction between these structural markers and process variables (such as Pb and compaction energy) to dictate the evolution of VV, VMA, and VFA. Utilizing Grey Relational Analysis (GRA), a sensitivity hierarchy is established to identify the principal regulators of the volumetric structure. Based on these insights, a ‘Robust Design’ methodology is developed, prioritizing the stabilization of the mineral framework through *D_c_* regulation. This study provides a theoretical and procedural foundation for shifting asphalt mixture design from empirical trial-and-error to a mechanism-based, precision-engineered methodology, offering a more stable and predictable alternative to current industry standards.

## 2. Theoretical Foundations and Parameter Definitions

### 2.1. Fractal Geometry and the Dual-Domain Fractal Model of Aggregate Gradation

Since Mandelbrot introduced fractal geometry, it has become a powerful tool for quantifying the irregular morphology and self-similarity of aggregate gradations. Asphalt mixture gradations typically exhibit a linear relationship in a double-logarithmic coordinate system, indicating that the particle size distribution possesses statistical fractal characteristics. This study builds on this framework by developing a dual-domain fractal model. The model decomposes the gradation into two processes: the construction of the coarse aggregate skeleton and the filling of the fine aggregate matrix. This approach establishes a theoretical foundation for subsequent performance prediction and optimization.

**Figure 1:**
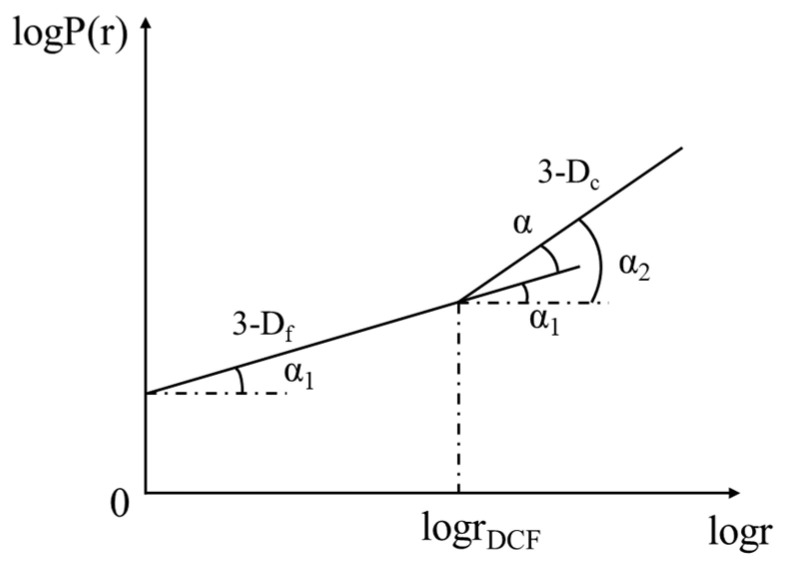
Fractal feature map of dual-domain fractal gradation.

The model uses a critical sieve size (*D_c_*, typically 2.36 mm or 4.75 mm) to partition the aggregate into two self-similar subsystems. As shown in Figure 1, the cumulative passing rate P(r) versus particle size r follows two distinct linear trends with different slopes in the log-log plot. These slopes determine the model’s primary parameters: the coarse fractal dimension (*D_c_*), representing skeleton stability, and the fine fractal dimension (*D_f_*), representing filling complexity.

#### 2.1.1. Coarse Gradation Fractal Dimension (*D_c_*)

The coarse fractal dimension (*D_c_*) is a key metric for evaluating the formation and stability of the coarse aggregate skeleton [33]. In this model, the primary role of coarse aggregates is to provide a structural framework; thus, *D_c_* reflects the particle distribution and stability of this framework. According to Zhang et al., the optimal range for *D_c_* is 2.3 to 2.6 [34,35]. A value below 2.3 suggests a loose skeleton with insufficient stability, while a *D_c_* exceeding 2.6 indicates an excess of smaller coarse particles. This can increase the risk of segregation, undermining structural continuity. Furthermore, Yu et al. found that mixtures with *D_c_* between 2.4 and 2.6 exhibit optimal skeleton stability, which significantly improves rutting resistance and high-temperature performance [36].

The formula for calculating the coarse gradation fractal dimension, *D_c_*, is expressed as:

(1) $$P(r)={\left(\frac{r}{r_{NMS}}\right)}^{3-D_{c}}P_{NMS},r\in [r_{DCF},r_{NMS}]$$

where $r_{NMS}$ is the nominal maximum aggregate size (mm); $r_{DCF}$ is the particle size at the coarse-fine boundary (mm); $P_{NMS}$ is the cumulative percentage passing the nominal maximum sieve (%); and *D_c_* is the coarse fractal dimension, which determines the skeletal structure.

#### 2.1.2. Fine Gradation Fractal Dimension (*D_f_*)

The fine fractal dimension (*D_f_*) characterizes the distribution of fine aggregates and the complexity of the filling phase. In the dual-domain model, fine aggregates primarily serve as filler material. Consequently, *D_f_* reflects both the distribution of fine particles and their ability to fill the voids within the coarse aggregate framework. Yu et al. [36] found that a *D_f_* range of 2.65 to 2.70 achieves optimal filling, significantly enhancing the compactness and stability of the asphalt mixture. Furthermore, Zhang et al. [34,35] observed that an increase in *D_f_* corresponds to a finer particle distribution: the 4.75–2.36 mm fraction decreases, while the 0.15–0.075 mm fraction significantly increases. This trend indicates that a higher *D_f_* enhances filling capacity; however, an excessive *D_f_* (>2.75) leads to an overly fine distribution, which may compromise the overall stability of the mixture.

The fine gradation fractal dimension (*D_f_*) is defined as:

(2) $$P(r)={\left(\frac{r}{r_{DCF}}\right)}^{3-D_{f}}P_{NMS},r\in [r_{\min},r_{DCF}]$$

where *r*_min_ is the minimum particle size (mm). In the majority of research concerning the fractal analysis of asphalt mixtures, *r*_min_ is conventionally assigned a constant value of 0.075 mm; and *D_f_* represents the fine aggregate fractal dimension, which characterizes the distribution of particles smaller than the domain separation point in the dual-domain fractal model.

#### 2.1.3. Coarse-to-Fine Aggregate Content Ratio (k)

The coarse-to-fine aggregate ratio (k) is defined as the mass ratio of coarse aggregate to fine aggregate, $k=\frac{m_{c}}{m_{f}}=1-\frac{P(r_{DCF})}{100}$. This ratio can also be expressed using the percentage of particles that pass through the sieve of a given size. As a fundamental parameter, k determines whether the coarse aggregate can form an efficient framework and whether the fine aggregate can achieve adequate filling. However, there is no consensus on the optimal value for k. This lack of agreement is primarily due to variations in coarse aggregate morphology (such as angularity and sphericity) and testing methodologies [37,38].

For instance, Zhang [39] suggested that the coarse aggregate skeleton should account for 72% of the total mass in gap-graded mixtures. Xu [40] proposed a range of 68% to 75% based on compressive strength assessments. Other researchers, focusing on the packing of spheres, recommended a fine aggregate content that fills approximately 26% of the porosity. These varying results indicate that k must be determined by integrating specific aggregate properties with the gradation design. In this study’s dual-domain fractal framework, k works in combination with *D_c_* and *D_f_* to provide precise control over the skeleton-filling structure.

#### 2.1.4. Fractal Evaluation Framework for Dual-Domain Fractal Gradation

The proposed evaluation framework is represented by the parameter set {*D_c_*, R_c_^2^; *D_f_*, R_f_^2^; k}. In this framework, R_c_^2^ and R_f_^2^ serve as statistical indicators of the “goodness of fit,” ensuring that the fractal model accurately represents the actual aggregate distribution. This system transitions gradation design from empirical observation to a mechanistic process by acknowledging the bimodal nature of asphalt mixtures. As emphasized by Tan [41], the mechanical response of a mixture is primarily dictated by its primary skeleton structure. By defining *D_c_* and *D_f_*, the model captures the distinct spatial distributions of the load-bearing and filling phases, respectively. Furthermore, the inclusion of the coarse-to-fine ratio (k) addresses the “interference effect”—a phenomenon where an imbalance between aggregate sizes leads to either skeleton dilation or insufficient densification [42].

To illustrate the advantages of this three-parameter approach over traditional discrete methods, a comparison is provided in Table 1.

The efficacy of this parametric characterization has been extensively validated. Research by Wang [3] identified strong linear relationships between fractal dimensions and volumetric features, while Sheng [43] developed quadratic regression models to link these metrics with dynamic stability. By consolidating these structural and process-related variables, the dual-domain fractal framework provides a robust theoretical basis for the precise optimization of asphalt mixture performance.

### 2.2. Fractal Characterization of Mesoscopic Void Structure

The compaction of asphalt mixtures creates a complex three-dimensional void network defined by the spatial arrangement and interlocking of aggregates [44]. This network determines the mixture’s moisture stability, durability, and mechanical performance. However, conventional volumetric indicators like air voids (VV) fail to capture the spatial complexity of the internal structure, such as void morphology and connectivity [45]. To address this, fractal theory offers a robust framework for quantifying these irregular void structures. It assumes that the spatial distribution and morphology of voids exhibit statistical self-similarity across multiple scales, allowing their geometric complexity to be characterized through fractal dimensions [46].

#### 2.2.1. Void Extraction Procedure Based on Digital Image Processing (DIP)

Digital Image Processing (DIP) was used to extract the mesoscopic void structure through three stages: image acquisition, preprocessing, and segmentation [47].

**Figure 2:**
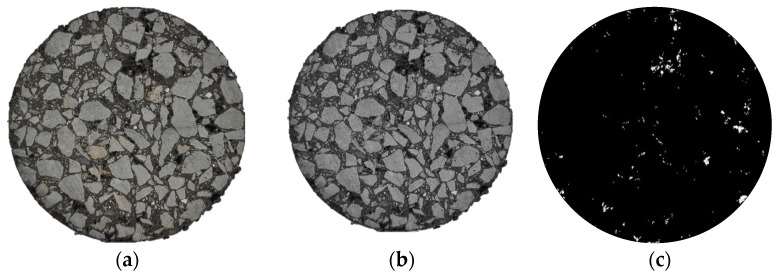
Digital image processing workflow for mesoscopic void extraction. (**a**) Original cross-sectional image; (**b**) preprocessed image with background removal; (**c**) binary image of void distribution using Otsu’s thresholding.

First, Marshall specimens were sliced vertically to obtain representative cross-sections. After polishing, high-resolution (6000 × 4000 pixels) images were captured under stable lighting to avoid shadows and distortion (Figure 2a).

The preprocessing stage entailed the conversion of the RGB images into 8-bit grayscale. A 3 × 3 median filter was applied in order to remove salt-and-pepper noise while preserving edge details. The area outside the specimen contour was replaced with pure white (gray value 255) in order to eliminate background interference and isolate the analytical domain (see Figure 2b).

Finally, void segmentation was performed using Otsu’s algorithm [48]. This method adaptively determines the optimal grayscale threshold by maximizing between-class variance, binarizing the image into voids (white pixels) and the solid phase (black pixels) (Figure 2c).

#### 2.2.2. Fractal Theory of Void Structure

The box-counting method is a widely adopted technique for the quantitative characterization of the morphological complexity and topological heterogeneity of asphalt mixture voids. In this study, the void area fractal dimension (D_V_) serves as a mesoscopic indicator to evaluate the spatial distribution and structural connectivity of the internal pore system.

**Figure 3:**
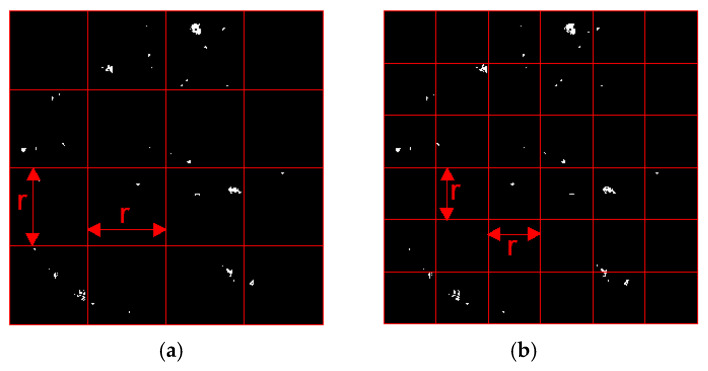
Schematic illustration of fractal dimension determination using the Box-counting method: (**a**) coarse grid; (**b**) finer grid [49].

The procedure involves overlaying a sequence of square grids with varying side lengths r onto the binarized cross-sectional image of the mixture. The number of cells, N(r), that contain at least one void pixel (the Region of Interest, ROI) is recorded for each scale. As illustrated in Figure 3, the observation scale is systematically refined from a coarse grid (Figure 3a) to a finer grid (Figure 3b), capturing the “evolution” of the void composition across different spatial resolutions. According to fractal geometry, the relationship between the grid size r and the box count N(r) follows a power-law distribution:

(3) $$N(r)\propto {\left(\frac{1}{r}\right)}^{D_{v}}$$

By plotting the recorded data on a log-log scale, with ln (1/r) as the independent variable and ln N(r) as the dependent variable, the fractal dimension D_V_ can be determined as the slope of the resulting linear regression line:

(4) $$Dv=\underset{r\to 0}{\lim}\frac{\ln N(r)}{\ln(1/r)}$$

In digital image analysis, the void pixels are isolated from the aggregate-binder background to form the ROI. While fractal dimensions can be categorized into contour and area types, this study specifically utilizes the void area fractal dimension. This choice is dictated by the need to characterize the overall mesoscopic occupancy and saturation of the void structure, providing a more comprehensive reflection of the mixture’s volumetric properties than a simple boundary-based analysis.

## 3. Materials and Methods

### 3.1. Materials

Basalt was used as the coarse aggregate, while limestone was used for the fine aggregate and mineral filler. Their density properties are listed in Table 2. The binder was Karamay 90# asphalt, which meets the requirements of the *Technical Specifications for Construction of Highway Asphalt Pavements* (JTG F40-2004) [50], as shown in Table 3.

For all subsequent experiments, asphalt mixture specimens were prepared at a standard compaction temperature of 150±5 °C in compliance with JTG 3410-2025 [51]. Specifically, aggregates were preheated at 180 °C for at least 4 h, and the binder was heated to 150 °C. The mixing process lasted for 180 s (including 90 s for aggregate-binder coating and an additional 90 s after adding mineral filler) to ensure uniform encapsulation. Specimens were then fabricated using a Marshall compactor, with 75 blows applied to each face to simulate heavy-traffic loading conditions and ensure a stable ‘skeleton-dense’ configuration.

### 3.2. Test Method

#### 3.2.1. Measurement and Calculation of Volume Parameters of Asphalt Mixture

Specimens were prepared as follows: To simulate field compaction and ensure data consistency, all specimens were fabricated using a Marshall compactor. The procedure followed method T 0702 in the Standard Test Methods of Bitumen and Bituminous Mixtures for Highway Engineering (JTG 3410-2025). Volumetric parameters were then determined according to method T 0705. Specifically, the Saturated Surface-Dry technique was used to measure the bulk specific gravity (G_mb_), calculated as the ratio of the specimen mass to its bulk volume:

(5) $$G_{mb}=m_{a}/(m_{f}-m_{w})$$

where m_a_ is the mass of the dry specimen in air, m_f_ is the saturated surface-dry mass, and m_w_ is the mass in water. The bulk density (ρ_b_) is then determined by ρ_b_ = G_mb_∗ρ_w_, using the water density at 25 °C (0.997 g/cm^3^).

To determine the air voids (VV), the theoretical maximum specific gravity (G_mm_) must first be established. According to the T 0711 method, G_mm_ represents the density of the mixture in a voidless state, calculated based on the effective specific gravity of the aggregate G_se_ and the specific gravity of the asphalt binder (G_b_):

(6) $$G_{mm}=\frac{100}{\frac{P_{s}}{G_{se}}+\frac{P_{a}}{G_{b}}}$$

Based on these foundational densities, the air voids (VV) are calculated as follows:

(7) $$VV=\left(1-\frac{G_{mb}}{G_{mm}}\right)\times 100$$

To evaluate the internal structure, the voids in mineral aggregate (VMA) are derived using the aggregate content (P_s_) and the bulk density of the combined aggregate (G_sb_) through the relation:

(8) $$VMA=\left(1-\frac{G_{mb}}{G_{sb}}\times P_{s}\right)\times 100$$

Subsequently, the asphalt saturation (VFA), representing the volume of VMA filled with effective asphalt:

(9) $$\mathrm{VFA}=\frac{\mathrm{VMA}-\mathrm{VV}}{\mathrm{VMA}}\times 100$$

Other indices incorporate: P_s_, the aggregate content derived from the sum of mass percentages of all mineral aggregate fractions relative to the total mixture mass (accurate to 0.1%); P_a_, the asphalt content representing the mass percentage of the binder; and P_ad_, the dosage of additives as a percentage of the total mass. These parameters are essential for assessing the “skeleton-dense” equilibrium state of the mixture and serve as vital benchmarks for the validation and calibration of the fractal model.

#### 3.2.2. Performance Characterization of Asphalt Mixtures

To evaluate the gradation design and validate the “skeleton-dense” structure, laboratory tests were performed following the *Standard Test Methods of Bitumen and Bituminous Mixtures for Highway Engineering* (JTG 3410-2025). These tests assessed three key performance indicators: moisture susceptibility, high-temperature stability, and low-temperature crack resistance.

(1) Moisture stability was evaluated using the Freeze–Thaw Splitting Test (T 0729). Specimens were divided into two groups: a control group and a conditioned group. The latter underwent a freeze–thaw cycle consisting of vacuum saturation, freezing at −18 °C for 16 h, and thawing in a 60 °C water bath for 24 h. The Tensile Strength Ratio (TSR) was calculated as the ratio of the split strength of the conditioned group ($R_{T2}$) to the unconditioned group ($R_{T1}$):

(10) $$\mathrm{TSR}=\frac{{\bar{R}}_{T2}}{{\bar{R}}_{T1}}\times 100$$

(2) High-temperature rutting resistance was evaluated using the Wheel Tracking Test (T 0719). Standard slab specimens (300 mm × 300 mm × 50 mm) were tested at 60 °C under a tire pressure of 0.7 MPa. The Dynamic Stability, defined as the number of wheel passes per millimeter of rutting deformation during the final 15 min of the one-hour test, was recorded as the primary indicator.
(3) The low-temperature performance was evaluated using the Three-Point Bending Test (T 0715) on small prismatic beams (250 mm × 300 mm × 35 mm). The specimens were loaded to failure at −10 °C with a strain rate of 50 mm/min. The Flexural-Tensile Strength (R_B_) and Maximum Flexural Strain ($\epsilon_{B}$) at failure were used to quantify the ductility and low-temperature resistance of the mixture.

#### 3.2.3. Extraction of Mesoscopic Void Structure

To ensure high-fidelity extraction of the internal void distribution for subsequent Digital Image Processing (DIP), this study combined precision sectioning with optimized surface preparation. Standard Marshall specimens served as the base samples. The sectioning was performed using a professional infrared-guided bridge-type stone cutting machine at a specialized facility (as shown in the experimental setup). This equipment provided the necessary stability and precision to maintain a consistent kerf width of approximately 0.5 mm, minimizing structural disturbance to the mineral skeleton.

A precision wet-cutting technology was employed, utilizing a continuous flow of water as a coolant and lubricant to prevent frictional heat from causing binder smearing—a common issue that can obscure mesoscopic voids. To ensure the accuracy of the subsequent examinations and prevent slurry (a mixture of cooling water and aggregate dust) from clogging the pores, a rigorous post-cutting cleaning protocol was implemented. Immediately after sectioning, all surfaces were subjected to high-pressure air cleaning followed by ultrasonic cleaning in a distilled water bath for 15 min to remove residual debris from the voids. Specimens were then oven-dried at a low temperature (30 °C) until a constant mass was reached, ensuring a high-contrast Region of Interest (ROI) for the binarization process.

**Figure 4:**
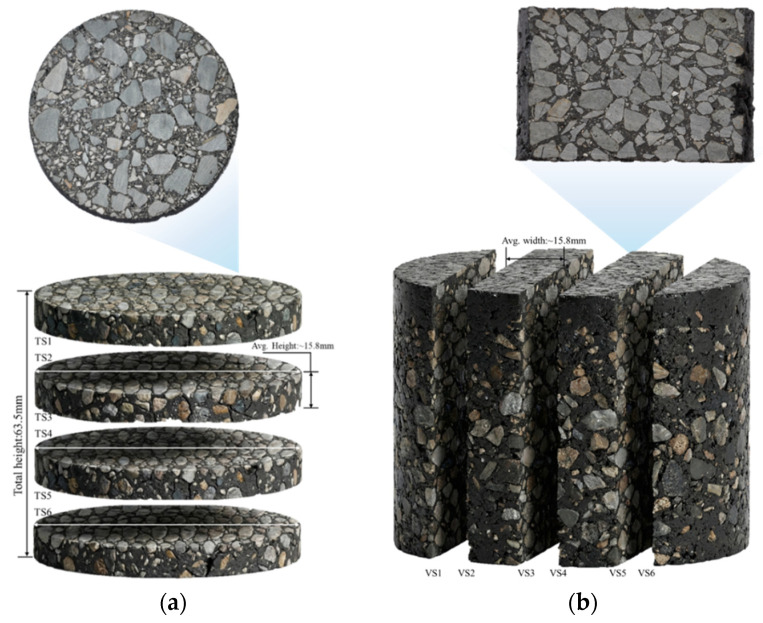
Schematic diagram of Marshall specimen sectioning: (**a**) horizontal view; (**b**) vertical view.

As shown in Figure 4a, the specimen was sliced horizontally at equal intervals along its height to produce six transverse sections (TS1 to TS6). For the vertical scheme (Figure 4b), the specimen was sectioned longitudinally along its diameter to obtain six vertical sections (VS1 to VS6). These sections provide a comprehensive dataset for characterizing the three-dimensional evolution of the void structure within the fractal-optimized mixtures.

#### 3.2.4. Calculation of Average Asphalt Film Thickness (AFT)

To further evaluate the durability and interfacial coating quality of the asphalt mixtures, the average asphalt film thickness (AFT) was introduced as a supplementary indicator. The AFT represents the theoretical thickness of the binder coating the aggregate particles and is a critical factor influencing the moisture resistance and long-term performance of the mixture. The calculation of AFT is performed in two steps: determining the total surface area of the mineral aggregate and calculating the film thickness based on the effective binder content.

The total surface area (SA) of the aggregate gradation is calculated using the surface area factor method recommended by the Asphalt Institute (MS-2) [52], as shown in Equation (11):

(11) $$SA=0.41+\sum_{i=1}^{n}\frac{C_{i}\times P_{i}}{100}$$

where SA is the total surface area ($m^{2}/kg$); C_i_ is the surface area factor for the i-th sieve size; and P_i_ is the percentage of aggregate passing the i-th sieve (%). The standard surface area factors utilized in this study are summarized in Table 4.

Subsequently, the AFT is determined by the relationship between the effective binder content and the total surface area, as expressed in Equation (12):

(12) $$\mathrm{AFT}=\frac{P_{be}}{SA\times \gamma_{b}\times (100-P_{b})}\times 10^{3}$$

where AFT is the average asphalt film thickness (μm); P_be_ is the effective asphalt content (%); P_b_ is the total asphalt content (%); and $\gamma_{b}$ is the relative density of the asphalt binder at 25 °C. This calculation framework ensures that the optimization of fractal parameters (*D_c_*, *D_f_*, and k) is evaluated not only from a volumetric perspective but also regarding the stability of the mastic-aggregate interface.

## 4. Influence of Gradation Fractal Parameters on Mixture Volumetric Properties

### 4.1. Influence of Coarse Fractal Dimension (D_c_) on Volumetric Properties

The skeletal effect of coarse aggregates is a decisive factor in the volumetric performance of asphalt mixtures. In a compacted state, the voids in the coarse aggregate (VCA) are filled by a mastic phase comprising fine aggregates, asphalt binder, and air. To isolate the specific impact of the coarse aggregate distribution, five gradations were designed with varying *D_c_* values, while the fine fractal dimension (*D_f_* = 2.70) and binder content (P_b_ = 5.0%) were held constant (Table 5). Marshall specimens were fabricated following the T 0702 protocol (75 blows per side); their volumetric properties are summarized in Table 6.

**Figure 5:**
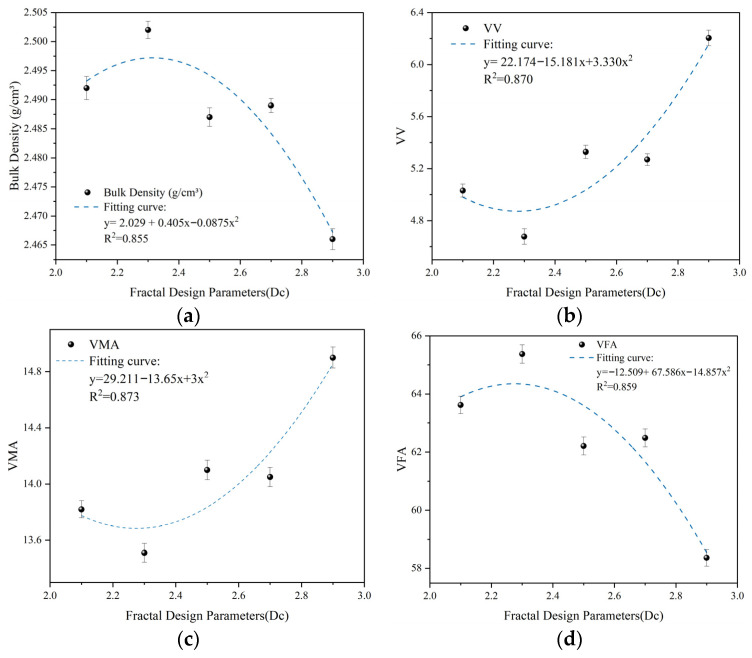
Relationship curves between coarse gradation fractal dimension (*D_c_*) and Marshall volumetric properties: (**a**) Bulk density variation curve; (**b**) Air voids (VV) variation curve; (**c**) VMA variation curve; (**d**) VFA variation curve.

Figure 5 illustrates the relationship between *D_c_* and Marshall volumetric properties. The data show that the volumetric indicators do not follow a simple linear relationship with *D_c_*; instead, they exhibit a parabolic trend. Specifically, specimen G2 (*D_c_* = 2.30) represents the inflection point of the gradation system, where the bulk density peaks at 2.502 g/cm^3^, while air voids (VV) and voids in mineral aggregate (VMA) reach their minimum values (4.677% and 13.51%, respectively). This indicates that at *D_c_* = 2.30, the coarse aggregate skeleton achieves optimal physical compatibility with the fine aggregate filler, approaching the ideal “skeleton-dense” state.

As *D_c_* increases from 2.5 to 2.9, the mixture density declines significantly. Starting from group G3, both VV and VMA reverse their downward trends; by group G5, VV increases to 6.204% and VMA rebounds to 14.90%. This phenomenon results from a mesoscopic “interference effect”: a higher *D_c_* indicates an increased proportion of smaller particles within the coarse fraction, which constricts the skeletal pore space. Given the constant fine aggregate and asphalt content, the restricted skeletal voids cannot accommodate the filler material. This triggers a “wedging action” that forces coarse particles apart to create additional space, leading to skeletal instability and an abnormal increase in void volume [53].

Comparing G5 and G2 reveals that although G5 has a more refined coarse fraction, its voids filled with asphalt (VFA) drop to a minimum of 58.36%. This suggests that the increased VMA at this stage consists primarily of voids generated by structural interference rather than effective asphalt-filled space.

As shown in Figure 5, the coefficients of determination (R^2^) for the fitted quadratic curves of all volumetric indicators exceed 0.85. Quadratic regression analysis confirms a significant relationship between *D_c_* and VV, with the minimum point at *D_c_* ≈ 2.30. Although the sample size is constrained by the intensive nature of laboratory mix preparation, the consistently high R^2^ values, coupled with the uniform parabolic trends across independent parameters (VV, VMA, and VFA), suggest that the dual-domain fractal dimension *D_c_* serves as a robust predictor for the evolution of the mixture’s internal structure. This value represents the theoretical threshold for the load-bearing capacity of the skeleton. Beyond this threshold, fine aggregate interference supersedes skeletal interlocking, compromising the volumetric stability of the mixture.

### 4.2. Influence of Fine Fractal Dimension (D_f_) and Volumetric Parameters

To evaluate the impact of fine aggregate gradation on volumetric performance, five experimental groups were designed based on the dual-domain fractal model (Table 7). While maintaining *D_c_* = 2.5, k = 0.7, and P_b_ = 5.0% as constant parameters, the fine aggregate fractal dimension (*D_f_*) was varied from 2.4 to 2.8.

**Figure 6:**
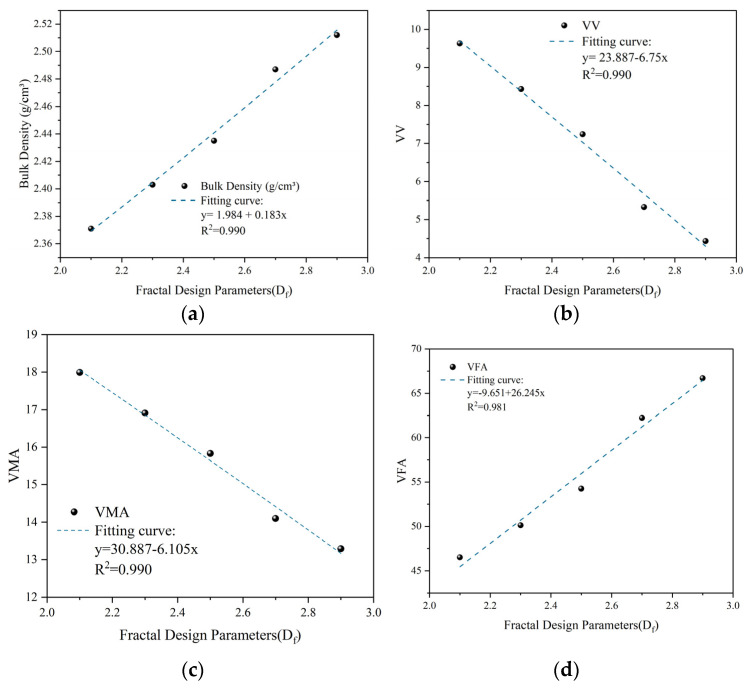
Relationship curves between fine gradation fractal dimension (*D_f_*) and Marshall volumetric properties: (**a**) Bulk density; (**b**) Air voids (VV); (**c**) VMA; (**d**) VFA.

As shown in Figure 6 and Table 8, *D_f_* exhibits a strong linear correlation with the mixture’s volumetric properties. The observed linear correlations remain valid within the stable range of the coarse aggregate skeleton. As *D_f_* approaches its physical packing limit, a non-linear transition might occur, but for the robust design range optimized here, the linear specification provides sufficient mechanistic insight. As *D_f_* increases, air voids (VV) and voids in mineral aggregate (VMA) show a monotonic decline—from 9.63% to 4.43% and 17.99% to 13.29%, respectively—while the voids filled with asphalt (VFA) increase from 46.5% to 66.7%.

These trends are consistent with the principles of mesoscopic packing: a higher *D_f_* indicates a greater proportion of fine fractions (0.075–0.6 mm), which effectively fill the interstices within the coarse skeleton according to particle packing theory. At a constant asphalt content, this reduction in VMA directly constrains the internal free space (VV) and enhances asphalt saturation (VFA). Consequently, the mixture transitions from a porous structure to a dense-saturated state. The observed linear correlations remain valid within the stable range of the coarse aggregate skeleton; while *D_f_* approaching its physical packing limit might trigger a non-linear transition, the linear specification provides sufficient mechanistic insight for the robust design range investigated in this study.

Physically, *D_f_* regulates the proliferation of fine particles within the mastic. An increase in *D_f_* implies a higher concentration of filler and fine sand, which leads to an exponential growth in the total specific surface area (SA). Under a constant binder dosage, this mechanism results in a redistribution of the asphalt binder, potentially thinning the average film thickness (AFT) and altering the interfacial bond strength.

### 4.3. Influence of Coarse-to-Fine Ratio (k) and Volumetric Parameters

The coarse-to-fine ratio (k), defined as the mass proportion of fine aggregate relative to the total aggregate mass, serves as a critical determinant of the mixture’s structural configuration. By maintaining constant fractal dimensions (*D_c_* = 2.5 and *D_f_* = 2.7), this section evaluates how variations in k alter the volumetric balance between the load-bearing skeleton and the filling phase (Table 9). Essentially, k acts as a structural switch, governing the transition between skeleton-dense and suspended-dense configurations by redistributing the spatial occupancy of aggregates.

**Figure 7:**
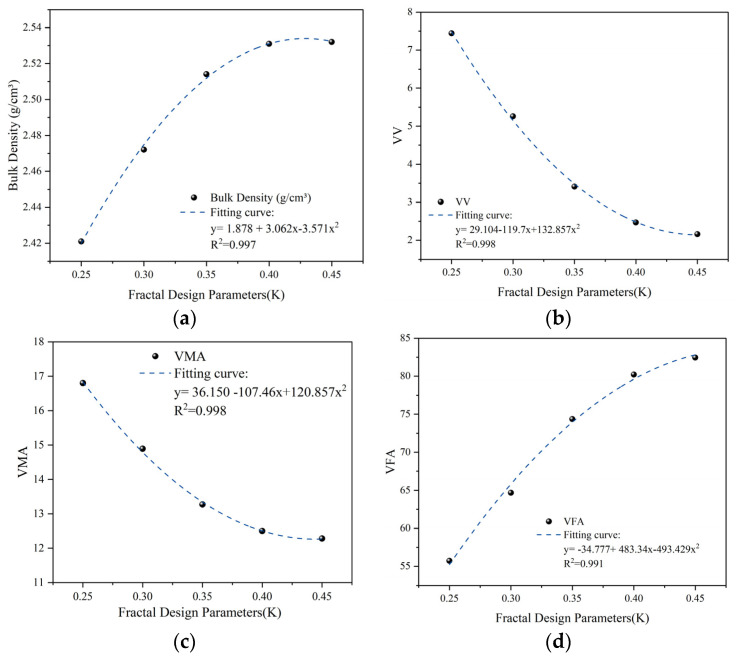
Relationship curves between coarse-to-fine ratio (k) and Marshall volumetric properties: (**a**) Bulk density; (**b**) Air voids (VV); (**c**) VMA; (**d**) VFA.

As shown in Table 10 and Figure 7, reducing k from 0.45 to 0.25 led to a pronounced monotonic change in all volumetric indicators. Specifically, the bulk specific gravity decreased steadily from 2.532 g/cm^3^ to 2.421 g/cm^3^. Concurrently, the air voids (VV) increased significantly from 2.16% to 7.44%, while the voids in mineral aggregate (VMA) rose from 12.28% to 16.80%. Accordingly, the voids filled with asphalt (VFA) showed a marked decline, dropping from 82.44% to 55.72%.

This phenomenon reveals a transition in the mesoscopic packing mechanism. At higher k values (e.g., 0.45), the mixture contains sufficient fine aggregate to overfill the coarse skeleton voids. In this state, the fine aggregate matrix not only occupies the mineral interstices but also causes the coarse aggregate particles to “suspend,” resulting in extremely low air voids (2.16%) and high saturation. While the mixture is highly dense, its rutting resistance may be compromised due to the loss of effective aggregate interlocking. As k decreases, the volume of fine aggregate becomes insufficient to fill the voids in the coarse aggregate (VCA). When k drops to 0.30 or 0.25, a network of interconnected, unfilled pores develops within the mineral system, leading to a rapid increase in VMA and VV. For instance, the VV of group G15 reached 7.44%, indicating a structural transition toward a porous skeletal configuration.

To quantify the sensitivity of volumetric indicators to the coarse-to-fine ratio (k), regression models were established (Figure 7). As shown in Figure 7, the relationship between k and the volumetric indicators (notably VV) yields an R^2^ exceeding 0.99. This near-ideal correlation is attributed to the fact that k serves as the fundamental mass-balance regulator between the skeletal and filling domains. Under the precision-controlled conditions of this study—where the fractal dimensions of the two domains are fixed—the volumetric filling efficiency becomes a direct function of the phase ratio k. This high degree of predictability confirms the validity of using k as a primary control parameter for precision air void engineering in arid-region pavement designs. In practical design, to balance density with skeletal interlocking, the VV is typically required to range between 3% and 5%. Based on the regression analysis, an ideal volumetric state is achieved when the k value is maintained within the range of 0.32 to 0.39.

The coarse-to-fine ratio k serves as a macro-regulator of the surface area budget. While *D_f_* dictates the ‘density’ of the surface area within the fine domain, k determines the total mass of the fine aggregate phase. Therefore, k acts as a secondary regulator of AFT by balancing the volume of the skeleton-forming coarse aggregates and the surface-area-dominating fine aggregates.

### 4.4. Decoupling the Effects of Gradation Parameters on D_V_

To elucidate the mesoscopic impact of gradation on the internal structure, this section shifts from macro-volumetric analysis to meso-scale morphological characterization. The focus is placed on correlating gradation parameters with the geometric complexity of air voids (D_V_). Based on the findings in Section 4.2, which identified *D_f_* as the primary driver of volumetric performance, *D_f_* is utilized here as the key independent variable. By analyzing its correlation with D_V_, this study aims to clarify how gradation design governs void “morphology” rather than mere “volume,” providing a multi-scale bridge between parametric design and structural performance.

**Figure 8:**
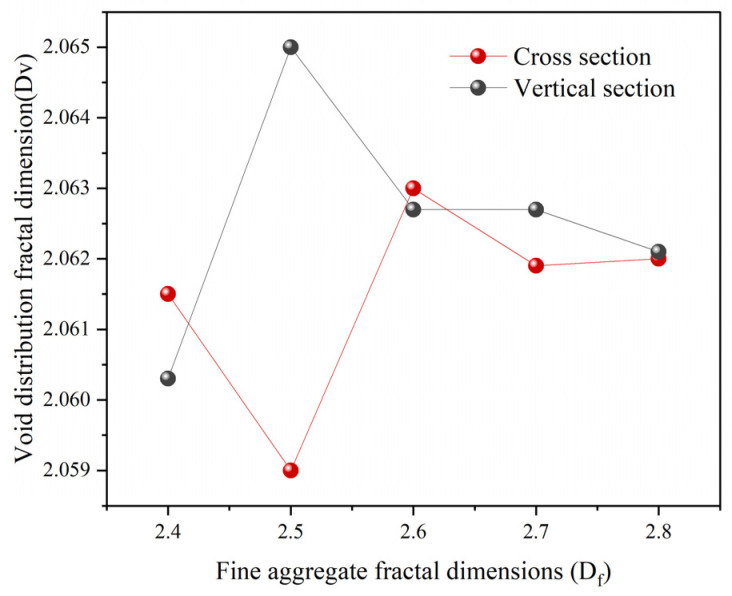
Relationship curves between fine aggregate fractal dimension (*D_f_*) and void distribution fractal dimension.

Based on the mean values of D_V_ recorded in Table 11, Figure 8 illustrates the variation in the void fractal dimension as a function of aggregate gradation.

The data reveal that D_V_ does not exhibit a monotonic trend as *D_f_* increases from 2.4 to 2.8. Instead, the values fluctuate marginally within a narrow range (2.059–2.065), maintaining a high degree of structural stability. This indicates that while variations in fine aggregate gradation significantly alter the macro-volumetric air voids (VV), they do not exert a systematic influence on the geometric complexity (D_V_) of the mesoscopic voids.

The observed insensitivity of D_V_ to *D_f_* suggests that the fractal characteristics of the internal void network are primarily governed by the contact force chain structure and the topological stability of the aggregate skeleton. These fundamental geometric properties appear to be independent of continuous gradation shifts. This finding confirms that adjusting the gradation is an effective strategy for regulating void volume without significantly altering the underlying void morphology, effectively decoupling these two structural attributes.

### 4.5. Mechanism Linking Fractal Parameters to Interfacial Coating Quality

The evolution of volumetric properties (VV, VMA, VFA) is intrinsically coupled with the evolution of the interfacial film thickness (AFT). Within the dual-domain fractal framework, the coarse fractal dimension *D_c_* defines the spatial volume of the interstitial voids (the ‘containers’), while *D_f_* and k define the surface area of the mineral phase (the ‘surface to be coated’). The mechanistic analysis reveals that an optimal gradation must achieve a ‘volumetric-interfacial balance’: it must provide a stable skeleton (*D_c_*) to maintain VMA while restricting the fine aggregate proliferation (*D_f_*) to ensure a sufficiently thick asphalt film for durability. This multi-scale interaction forms the theoretical basis for the gradation optimization method discussed in Section 5.

## 5. Sensitivity Analysis of Volumetric Properties and Gradation Optimization

This section quantifies the response of asphalt mixture volumetric properties to various design inputs, establishing a parameter influence hierarchy based on the preceding experimental results.

### 5.1. Orthogonal Experimental Design and Volumetric Responses

The air void content (VV) is a critical indicator of pavement performance, governed by three primary categories: aggregate gradation, binder content (P_b_), and compaction effort. Within the dual-domain fractal framework, gradation is uniquely defined by *D_c_*, *D_f_*, and k. However, in practice, the volumetric structure results from the synergy between material composition and processing conditions. While P_b_ influences the lubrication and filling efficacy of the mastic, the compaction effort determines the ultimate packing density.

To systematically decouple the independent and interactive effects of fractal characteristics and process parameters, this study integrates *D_c_*, *D_f_*, k, P_b_, and the number of compactions blows into an integrated analytical framework. An orthogonal experimental design was employed to evaluate the influence patterns of these five factors on VV (Table 12).

Using the *D_c_*, *D_f_*, and k values assigned to each group, the resultant gradations were calculated (Table 13). Marshall specimens were prepared based on the specified binder contents (P_b_) and compaction effort, followed by VV measurements. Through range analysis and Grey Relational Analysis (GRA), this study evaluates the sensitivity of VV to each control parameter and identifies the dominant influencing factors. These results provide a robust foundation for subsequent quantitative sensitivity analysis and multi-objective gradation optimization.

### 5.2. Grey Relational Analysis (GRA) of Volumetric Indicators

The volumetric structure of an asphalt mixture results from the synergy between gradation design, binder content (P_b_), and compaction effort (N). To quantify the impact of the dual-domain fractal parameters (*D_c_*, *D_f_*, k) alongside engineering factors (P_b,_ N) on volumetric responses, Grey Relational Analysis (GRA) was performed. Based on the $L_{25}(5^{6})$ orthogonal experimental results (Table 14), the sensitivity of these five factors was ranked to identify the dominant parameters for gradation optimization.

The specific configurations of the $L_{25}(5^{6})$ orthogonal array, integrating both fractal parameters (*D_c_*, *D_f_*, k) and engineering variables (P_b_, N), are detailed in Table 13. This comprehensive matrix recorded the VV responses across 25 distinct experimental conditions, providing the necessary data for subsequent range analysis and Grey Relational Grade (GRG) calculations. To ensure statistical reliability, each VV value represents the average of three replicate specimens.

#### 5.2.1. Grey Relational Analysis (GRA) Framework

(1) Reference and Comparative Sequences

To evaluate the influence of each parameter, we define the Reference Sequence (target responses) and Comparative Sequences (influencing factors). Let the experimental results for a specific volumetric indicator (e.g., VV or VMA) be denoted as X_0_:

(13) $$X_{0}=\{X_{0}(1),X_{0}(2),\ldots ,X_{0}(n)\}$$

The influencing factors—including *D_c_*, *D_f_*, k, P_b_, and N—constitute the Comparative Sequences X_i_:

(14) $$X_{i}=\{X_{i}(1),X_{i}(2),\ldots ,X_{i}(n)\},\;i=1,2,\ldots ,m$$

where n = 25 (experimental groups) and m = 5 (influencing factors).

(2) Data Normalization

Because the investigated factors have different units and magnitudes (e.g., N is 10^2^ while k is 10^−1^), the raw data must be normalized. This study employs min-max scaling to map the original values into the dimensionless interval [0, 1]:

(15) $${x^{\prime }}_{i}(k)=\frac{x_{i}(k)-\min X_{i}(k)}{\max X_{i}(k)-\min X_{i}(k)}$$

where ${x^{\prime }}_{i}(k)$ is the standardized data, and $\min X_{i}$ and $\max X_{i}$ are the minimum and maximum values of the sequence $X_{i}$, respectively.

(3) Grey Relational Coefficient

The absolute difference $\Delta_{i}(k)$ between the comparative sequence x_i_ and the reference sequence x_0_ at the k-th trial is:

(16) $$\Delta_{i}(k)=\left|x_{0}(k)-x_{i}(k)\right|$$

The Grey Relational Coefficient $\xi_{i}(k)$ is then calculated as:

(17) $$\xi_{i}(k)=\frac{\Delta_{\min}+\rho \Delta_{\max}}{\Delta_{i}(k)+\rho \Delta_{\max}}$$

where $\Delta_{\min}$ and $\Delta_{\max}$ are the global minimum and maximum absolute differences. The distinguishing coefficient ρ is set to 0.5 to ensure adequate resolution between the factors.

(4) Grey Relational Grade (G_i_)

The Grey Relational Grade (GRG) represents the arithmetic mean of the relational coefficients across all trials, characterizing the overall correlation between the factors and the target indicator:

(18) $$G_{i}=\frac{1}{n}\sum_{k=1}^{n}\xi_{i}(k)$$

A $G_{i}$ value closer to 1 signifies that the volumetric indicator is more sensitive to that specific factor.

#### 5.2.2. Discussion of Sensitivity Ranking Results

**Figure 9:**
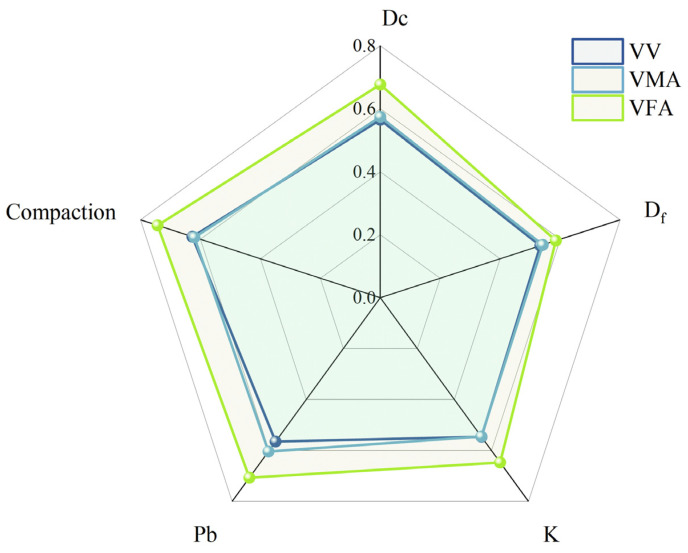
Radar chart of grey relational grades between design factors and volumetric indicators (Note: The sensitivity rankings of compaction cycles are based on a constant optimal compaction temperature; deviations in binder viscosity will significantly alter the energy required for densification.).

According to the Grey Relational Analysis (GRA) results presented in Table 15 and Figure 9, the sensitivity of asphalt mixture volumetric properties follows a distinct hierarchy that informs a mechanistic approach to mix design.

Among all investigated indicators, the compaction effort (N) demonstrated the highest relational grades, specifically 0.627 for air voids (VV), 0.620 for voids in mineral aggregate (VMA), and 0.743 for voids filled with asphalt (VFA). These results underscore that external compaction energy is the primary factor governing the final volumetric framework. Notably, the high synchronization between compaction effort and binder content (P_b_) regarding the VFA (with grades of 0.743 and 0.707, respectively) reflects their fundamental physical intercorrelation. Under the constant standard compaction temperatures maintained in this study, the asphalt binder serves as a critical lubricating agent that reduces internal friction between aggregates, while the compaction cycles provide the external energy required for structural rearrangement. This synergy implies that the efficiency of “Process Control” is intrinsically coupled with the rheological state of the phase-filling medium, which together dictate the densification degree of the mixture.

Regarding internal material variables, the volumetric indicators demonstrate distinct functional differentiation. For VMA, the relational grade of the coarse aggregate fractal dimension *D_c_* (0.604) exceeds that of the binder content P_b_ (0.574), confirming that the geometric complexity of the mineral skeleton is a more dominant driver of interstitial volume than asphalt dosage. This finding embodies the “skeleton-dominant” principle, where the distribution of coarse aggregates establishes the primary structural framework of the mixture. Conversely, for the VFA indicator, the sensitivity to P_b_ (0.707) is significantly higher than to *D_c_* (0.677), identifying the binder as the primary phase-filling medium within the compacted matrix.

For air voids (VV), the relational grades for *D_c_* and P_b_ are nearly identical at 0.565, revealing two parallel pathways for achieving equivalent densification: optimizing skeletal interlocking or adjusting binder content. This provides engineers with flexible options for mix design optimization depending on material availability or performance requirements. Further analysis of the fractal parameters reveals a consistent hierarchy where *D_c_* ranks higher than the fine aggregate fractal dimension *D_f_*, while the coarse-to-fine ratio k serves as a transitional regulatory parameter with an influence falling between the two dimensions. This stable hierarchy reinforces the central role of coarse aggregate distribution in controlling macro-volumetric parameters.

In summary, the synthesis of data from Table 14 and Figure 9 establishes a four-tier hierarchical design logic: Process Control (Compaction) > Skeleton Regulation (*D_c_*) > Phase Filling (P_b_) > Gradation Fine-tuning (k, *D_f_*). This framework provides a theoretical foundation for transitioning from empirical trial-and-error toward a mechanism-clear design methodology that is resilient to construction fluctuations. Such a hierarchy is particularly vital for engineering projects in challenging environments, such as the cold and arid regions of Xinjiang, where it can enhance the mixture’s resistance to environmental extremes and construction variability. This multi-scale sensitivity evaluation not only strengthens the understanding of internal structural evolution but also offers procedural guidance for the refined design of asphalt pavements in arid regions.

### 5.3. Gradation Optimization Workflow Based on Sensitivity Hierarchy

**Figure 10:**
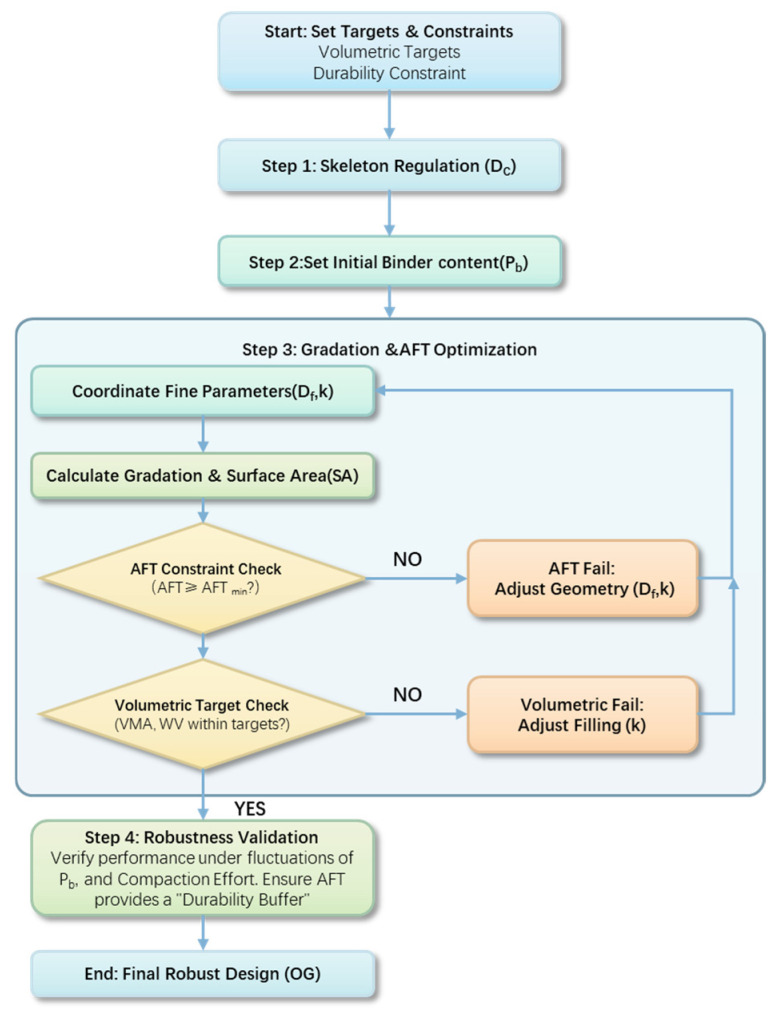
Flowchart of the robust gradation optimization workflow based on dual-domain fractal parameters and the mandatory AFT boundary condition.

Building upon the sensitivity analysis conclusions, this section proposes a gradation optimization workflow with fractal parameters as the regulatory core to achieve a robust asphalt mixture design. The objective is to ensure stable volumetric parameters and reduced sensitivity to fluctuations in construction conditions by adhering to the fundamental principle of Process Control (Compaction) > Skeleton Regulation (*D_c_*) > Phase Filling (P_b_) > Gradation Fine-tuning (k, *D_f_*) with AFT constraint. The comprehensive optimization workflow, integrated with a mandatory durability boundary condition, is illustrated in Figure 10. Within this framework, “stability” denotes that the critical volumetric indicators, such as VV and VMA, remain within target intervals, while durability is enforced by maintaining the average asphalt film thickness (AFT) above a mandatory threshold (e.g., 8 μm).

The optimization process begins with the priority determination of the coarse aggregate fractal dimension (*D_c_*) to construct a stable initial skeleton, leveraging the high degree of control *D_c_* exerts over the VMA. Once the skeleton is established and the target binder content (P_b_) is set, the workflow enters the filling and fine-tuning phase. In this stage, the fine aggregate fractal dimension (*D_f_*) and the coarse-to-fine ratio (k) are coordinated not only to fill the skeletal voids but also to regulate the total specific surface area (SA). To ensure interfacial durability, the AFT is integrated as a mandatory boundary condition. If the combination of *D_f_* and k results in an excessive SA that causes the AFT to drop below the safety limit, the gradation parameters must be re-adjusted by reducing *D_f_* or increasing k to streamline the fine aggregate fraction.

Finally, a robustness validation and reliability check are conducted to verify the design results within the expected variation ranges of the asphalt-aggregate ratio and compaction effort. The inclusion of the AFT constraint ensures that the “Robust Design” is characterized by a “Durability Buffer”—where the optimized gradation (OG) provides a sufficiently thick asphalt film to resist environmental stressors. This integrated approach ensures the design’s tolerance and reliability during actual field construction, bridging the gap between mechanical stability and long-term pavement durability in cold and arid regions.

### 5.4. Validation of Optimized Gradation: Comparison with Conventional Design Methods

Building upon the established optimization workflow, this section evaluates the effectiveness of the fractal design procedure by comparing the optimized gradation with traditional design methods.

#### 5.4.1. Comparative Experimental Scheme

Two sets of gradations were established to conduct a comparative experimental analysis. The Experimental Group (OG) utilized the “Fractal Optimized Gradation” designed according to the workflow detailed in Section 5.3. For this group, *D_c_* = 2.3 was selected from the candidate range (2.30 to 2.70) as it yielded a predicted VMA closest to the center of the target range (13.5–15.5%). Subsequently, the combination of *D_f_* = 2.55 and k = 0.84 was identified to satisfy the requirements for predicted VV (4%) and VFA (65.0–75.0%).

**Figure 11:**
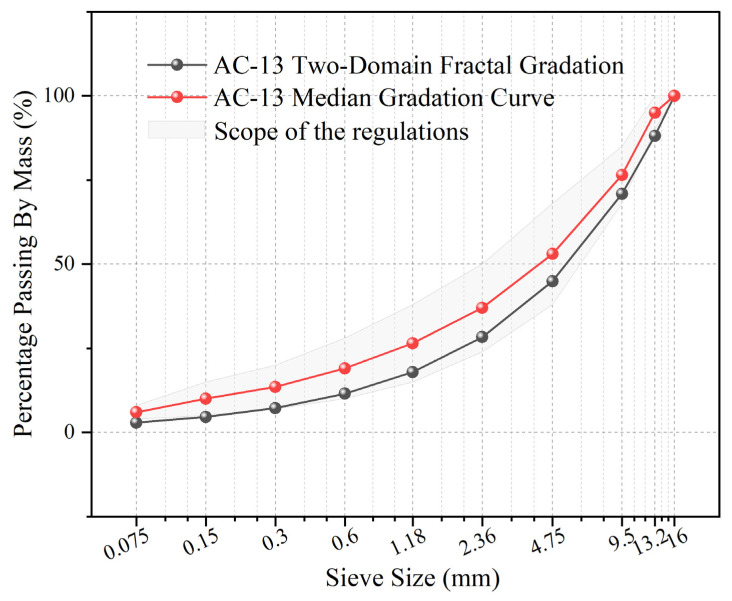
Comparison between the dual-domain fractal gradation curve and the standard median gradation curve for AC-13.

In contrast, the Control Group (CG) represented the traditional empirical gradation, utilizing the median values of the AC-13 gradation range specified in the *Technical Specifications for Construction of Highway Asphalt Pavements* (JTG F40-2004). To ensure a rigorous comparison and isolate the influence of the gradation structure, both groups utilized identical asphalt binders at the same median asphalt-aggregate ratio and the same initial number of compaction passes. The comparative gradation curves for both groups are illustrated in Figure 11.

#### 5.4.2. Comparative Analysis of Volumetric Stability and Interfacial Durability

The comparative results of the volumetric and interfacial indicators for the Optimized Gradation (OG) and the Control Group (CG) are summarized in Table 16. To provide a more mechanistic evaluation, the average asphalt film thickness (AFT) was calculated based on the total specific surface area (SA) using the Asphalt Institute MS-2 method.

As shown in Table 16, the OG group achieved a VV of 4.35% and a VMA of 14.53%, both of which fall within the ideal design range for AC-13 mixtures. While the CG group exhibited a higher bulk density and lower air voids, the core of the stability verification lies in the response of these indicators to external energy fluctuations and their long-term durability. This comparative analysis highlights the structural and interfacial superiority of the fractal-optimized approach through four key dimensions:

First, the OG group demonstrates enhanced skeletal robustness. According to the sensitivity rankings identified in Section 5.2, the coarse aggregate fractal dimension (*D_c_*) is the primary factor controlling the VMA. By prioritizing the optimization of *D_c_* at 2.30, the OG group establishes a more robust mineral skeleton. This ensures that inter-particle rearrangement is significantly constrained when compaction effort deviates from laboratory protocols, thereby simulating construction site variations more reliably.

Second, the fractal optimization design successfully mitigates compaction sensitivity. Although the CG mixture is denser, its reliance on a “suspended-dense” logic makes its VV more susceptible to over-compaction or under-compaction. In contrast, the OG mixture, characterized by its “skeleton-dense” configuration, demonstrates a broader “process window” that maintains the target volumetric state despite construction fluctuations.

Third, the OG group exhibits significantly superior interfacial durability. A critical finding in this comparative study is that the OG mixture achieves an AFT of 15.03 μm, which is approximately 75% thicker than that of the CG mixture (8.61 μm). This improvement is achieved without increasing the asphalt dosage (P_b_) but through the strategic regulation of the fine aggregate fractal dimension (*D_f_*) and the coarse-to-fine ratio (k). By reducing the excessive fine aggregate content, the total specific surface area (SA) is reduced from 5.93 m^2^/kg to 3.40 m^2^/kg. The resulting thicker asphalt film provides a more robust protective barrier for the aggregate-mastic interface, which is essential for resisting moisture damage and aging in the extreme environments of the Xinjiang region.

Finally, the OG group demonstrates superior tolerance to material variations. The VFA of the OG group (66.66%) is more centrally located within the typical specification range (65–75%) compared to the CG group (72.47%). This creates a “buffering capacity” that allows the mixture to accommodate variations in asphalt dosage and compaction energy without risking sudden drops in air voids, thus enhancing resistance to rutting and bleeding while maintaining sufficient film thickness for durability.

In conclusion, this comparative analysis validates that the fractal-optimized gradation (OG) transcends the limitations of traditional empirical methods. By anchoring the design in the most sensitive fractal parameters and incorporating AFT as a durability constraint, the proposed workflow achieves a “Robust Design” that effectively bridges the gap between laboratory targets and field performance in cold and arid regions.

#### 5.4.3. Preliminary Evaluation and Mechanical Analysis of Pavement Performance

The pavement performance results for the OG and CG provide a preliminary indication of the potential advantages offered by the fractal-based design. Regarding high-temperature rutting resistance, the data in Table 17 indicate that the OG mixture possesses a significantly higher dynamic stability (DS) of 2617 times/mm compared to 1230 times/mm for the conventional CG. This improvement is consistent with the prioritized optimization of *D_c_*, which was identified as a primary regulator of the mineral skeleton. From a mechanical perspective, engineering a robust skeleton-dense structure enhances the interlocking between coarse particles, thereby contributing to superior resistance against shear deformation at elevated temperatures.

Regarding low-temperature performance at −10 °C, the results show that the OG mixture maintains a comparable level of flexural response to the CG mixture. While the CG group exhibits slightly higher failure stress (10.02 MPa) and strain (2.712), the differences are marginal (approximately 0.6% and 2.0%, respectively). The slight reduction in peak strain in the OG group is a natural consequence of the reinforced mineral skeleton (*D_c_*), which increases the stiffness of the mixture. However, it is essential to note that the OG mixture offers a superior “Durability Buffer” due to its significantly higher Average Film Thickness (AFT = 15.03 μm vs. 8.61 μm), as discussed in Section 5.4.2. This thicker film ensures better protection against binder aging and moisture infiltration, which are primary drivers of cracking in the cold and arid environments of Xinjiang.

Similarly, the water stability results suggest that the OG maintains reliable moisture resistance at 87.6%, compared to 86.5% for the CG. This supports the objective of achieving a more stable void morphology through fractal optimization. In summary, the performance data suggest that by anchoring the design in sensitive fractal parameters, it is possible to achieve a better balance—significantly enhancing rutting resistance and interfacial durability while maintaining adequate low-temperature properties. These findings provide a promising basis for the “Robust Design” approach.

### 5.5. Discussion

#### 5.5.1. The “Skeletal-Infilling” Mechanism in Dual-Domain Fractals

The transition from a single fractal model to a dual-domain framework (*D_c_* and *D_f_*) represents more than a mathematical refinement; it reflects the physical reality of asphalt mixtures as a multi-phase composite [13]. The results in Section 4 indicate that *D_c_* primarily dictates the skeleton’s rigidity, while *D_f_* governs the mastic filling. This “decoupling” of coarse and fine aggregate roles suggests that the volumetric stability of the mixture is not a product of the entire gradation’s entropy, but rather a hierarchical coordination of different size scales [54]. By isolating *D_c_* as the “Skeleton Regulator,” we can engineer a stone-on-stone contact structure that remains resilient even when the binder content (P_b_) fluctuates during high-temperature service or intense construction cycles. This aligns with the findings of Lugo et al. [55], who noted that the interlocking efficiency of coarse aggregates is independent of the fine aggregate distribution, provided the latter does not “over-fill” the primary voids. The dual-domain approach provides a quantitative tool to prevent such interference, ensuring structural stability under heavy loading.

#### 5.5.2. Synergy Between Film Thickness and Interfacial Durability

The significant increase in average film thickness (AFT) in the Optimized Gradation (OG) group reveals the efficiency of the dual-domain approach in redistributing the asphalt mastic. Unlike conventional empirical methods that often lead to “dry” or over-filled spots, the fractal-based k-ratio ensures that the mastic is distributed more uniformly across the massive surface area of the fine aggregates [56].

This increased AFT is not merely a numerical gain; it provides a vital sacrificial layer against the intense UV radiation and oxidative aging characteristic of cold and arid climates, such as the Xinjiang region [57,58]. A thicker asphalt film has been shown to significantly delay the rate of binder hardening by limiting oxygen diffusion into the aggregate-binder interface [59]. The synergy between a robust *D_c_* skeleton and a thick *D_f_*-driven film essentially creates a “double-armored” structure that balances mechanical rutting resistance with long-term chemical durability.

## 6. Conclusions and Suggestions

### 6.1. Conclusions

This study established a comprehensive dual-domain fractal framework to characterize the internal architecture of asphalt mixtures, providing a mechanistic link between multiscale structural parameters and macroscopic performance. The main conclusions are as follows:

Mechanistic Characterization: The proposed dual-domain model, integrating the coarse aggregate fractal dimension (*D_c_*), the fine aggregate fractal dimension (*D_f_*), and the coarse-to-fine ratio (k), serves as a precise mathematical instrument for decoupling the structural roles of the load-bearing skeleton and the interstitial filling phase. This allows for a more granular regulation of the “skeleton-dense” state compared to traditional empirical methods.

Sensitivity Hierarchy and Design Robustness: Through Grey Relational Analysis (GRA), a clear control hierarchy for mixture design was established: Process Control (Compaction) > Skeleton Regulation (*D_c_*) > Phase Filling (P_b_) > Gradation Fine-tuning (k, *D_f_*). By prioritizing *D_c_* to anchor the mineral framework, the resulting “Robust Design” exhibits significantly reduced sensitivity to construction fluctuations, ensuring volumetric stability under varying field conditions.

Significant Performance Enhancement: The practical superiority of this approach was validated through mechanical and functional testing. The optimized gradation (OG) achieved a 112% increase in high-temperature dynamic stability (from 1230 to 2617 times/mm) by engineering a more robust interlocking skeleton.

Interfacial Durability and Functional Balance: A critical functional improvement is the 75% increase in average film thickness (AFT), rising from 8.61 μm to 15.03 μm without increasing the binder content. This provides a substantial “durability buffer” against moisture damage and aging, which is vital for pavements in the extreme cold and arid environments of Xinjiang. Furthermore, the OG mixture maintains comparable low-temperature cracking resistance and moisture stability to conventional designs, achieving a superior overall performance balance.

### 6.2. Suggestions

Based on the current findings, the following suggestions are proposed for future development:

Material Versatility: Future efforts should verify the fractal optimization parameters (*D_c_*, *D_f_*, k) across a broader range of material systems, such as Stone Matrix Asphalt (SMA) and rubber-modified binders, to enhance the universality of the “Robust Design” framework.

Field-scale Validation: Transitioning from laboratory specimens to large-scale field trials is essential to confirm the long-term efficacy of the dual-domain framework under real-world traffic loading and environmental aging.

Intelligent Optimization: Integrating these deterministic fractal parameters into advanced computational frameworks, such as machine learning algorithms or digital twin models, could automate the optimization process and promote high-precision pavement engineering.

## Figures and Tables

**Table 1:** Comparison between Conventional and Three-Parameter Fractal Gradation Design.

Feature	Conventional Methods	Three-Parameter Fractal Method	Advantage
Description	Discrete passing percentages at specific sieves	Continuous fractal dimensions (*D_c_*, *D_f_*)	Captures the complexity of the entire particle size distribution
Structural Logic	Vague distinction between coarse and fine aggregates	Explicit “Skeleton-Filling” dual-domain model	Aligns with the actual load-bearing structure of the mixture
Design Control	Empirical matching of control points	Parametric control through the coupling factor (k)	Enhances precision and reduces experimental workload
Optimization	Trial-and-error based on discrete intervals	Analytical optimization based on parameter sensitivity	Facilitates scientific and reproducible gradation design

**Table 2:** Test results of aggregate density.

Density Parameters (g/cm^3^)	Sieve Size (mm)									
	13.2	9.5	4.75	2.36	1.18	0.6	0.3	0.15	0.075	Mineral Filler
	Basalt			Limestone						
$\rho_{b}$	2.747	2.742	2.843	2.686	2.677	2.678	2.677	2.686	2.695	2.729
$\rho_{a}$	2.914	2.917	3.049	——	——	——	——	——	——	——

Note: $\rho_{a}$ denotes the apparent density of the coarse and fine aggregates, and $\rho_{b}$ represents the bulk density of coarse aggregates.

**Table 3:** Basic properties of Karamay 90# asphalt.

Sample	Penetration (25 °C, 0.1 mm)	Softening Point (°C)	Ductility (10 °C, cm)	Ductility (15 °C, cm)	Density (g/cm^3^)
Karamay 90#	88	47	>150	>150	0.9815
Test method	T0604-2011	T0606-2011	T0605-2011	T0605-2011	T0603-2025

**Table 4:** Surface area factors for mineral aggregates (MS-2).

Sieve Size (mm)	4.75	2.36	1.18	0.60	0.30	0.15	0.075
Surface Area Factor ($C_{i},m^{2}/\mathrm{kg}$)	0.41	0.82	1.64	2.87	6.14	12.29	32.77

**Table 5:** Composition of experimental gradations with varying coarse gradation fractal dimensions (*D_c_*).

Gradation No.	Fractal Design Parameters	Percent Passing by Sieve Size (mm) (%)									
	(*D_c_*, *D_f_*)	16	13.2	9.5	4.75	2.36	1.18	0.6	0.3	0.15	0.075
G1	(2.1, 2.7)	100	95	67.31	30	24.32	19.76	16.13	13.1	10.64	8.64
G2	(2.3, 2.7)	100	95	68.84	30	24.32	19.76	16.13	13.1	10.64	8.64
G3	(2.5, 2.7)	100	95	70.36	30	24.32	19.76	16.13	13.1	10.64	8.64
G4	(2.7, 2.7)	100	95	71.87	30	24.32	19.76	16.13	13.1	10.64	8.64
G5	(2.9, 2.7)	100	95	73.35	30	24.32	19.76	16.13	13.1	10.64	8.64

**Table 6:** Marshall volumetric properties of asphalt mixtures with various *D_c_* values.

Gradation No.	Fractal Design Parameters	Volumetric Properties			
	(*D_c_*, *D_f_*)	Bulk Density (g/cm^3^)	VV	VMA	VFA
G1	(2.1, 2.7)	2.492	5.030	13.82	63.62
G2	(2.3, 2.7)	2.502	4.677	13.51	65.37
G3	(2.5, 2.7)	2.487	5.328	14.10	62.21
G4	(2.7, 2.7)	2.489	5.270	14.05	62.49
G5	(2.9, 2.7)	2.466	6.204	14.90	58.36

**Table 7:** Composition of experimental gradations with varying fine gradation fractal dimensions (*D_f_*).

Gradation No.	Fractal Design Parameters	Percent Passing by Sieve Size (mm) (%)									
	(*D_c_*, *D_f_*)	16	13.2	9.5	4.75	2.36	1.18	0.6	0.3	0.15	0.075
G6	(2.5, 2.4)	100	95	70.36	30	19.72	13.01	8.67	5.72	3.77	2.49
G7	(2.5, 2.5)	100	95	70.36	30	21.15	14.95	10.66	7.54	5.33	3.77
G8	(2.5, 2.6)	100	95	70.36	30	22.68	17.19	13.12	9.95	7.54	5.72
G9	(2.5, 2.7)	100	95	70.36	30	24.32	19.75	16.12	13.09	10.63	8.63
G10	(2.5, 2.8)	100	95	70.36	30	26.08	22.7	19.83	17.26	15.02	13.08

**Table 8:** Marshall volumetric properties of asphalt mixtures with various *D_f_* values.

Gradation No.	Fractal Design Parameters	Volumetric Properties			
	(*D_c_*, *D_f_*)	Bulk Density (g/cm^3^)	VV	VMA	VFA
G6	(2.5, 2.4)	2.371	9.63	17.99	46.5
G7	(2.5, 2.5)	2.403	8.43	16.91	50.13
G8	(2.5, 2.6)	2.435	7.24	15.83	54.26
G9	(2.5, 2.7)	2.487	5.33	14.1	62.22
G10	(2.5, 2.8)	2.512	4.43	13.29	66.7

**Table 9:** Composition of experimental gradations with variable ratios of coarse and fine grading quantities (k).

Gradation No.	Ratio of Coarse and Fine Grading Quantities	Percent Passing by Sieve Size (mm) (%)									
	k(P(4.75))	16	13.2	9.5	4.75	2.36	1.18	0.6	0.3	0.15	0.075
G11	0.45	100	95	76.05	45	34.02	25.78	19.67	14.91	11.3	8.57
G12	0.40	100	95	74.15	40	30.24	22.92	17.49	13.26	10.05	7.62
G13	0.35	100	95	72.26	35	26.46	20.05	15.3	11.6	8.79	6.67
G14	0.30	100	95	70.36	30	22.68	17.19	13.12	9.95	7.54	5.72
G15	0.25	100	95	68.47	25	18.9	14.32	10.92	8.27	6.26	4.74

**Table 10:** Marshall volumetric properties of asphalt mixtures with various k values.

Gradation No.	k(P(4.75))	Gradation No.			
		Bulk Density (g/cm^3^)	VV	VMA	VFA
G11	0.45	2.532	2.16	12.28	82.44
G12	0.40	2.531	2.47	12.5	80.21
G13	0.35	2.514	3.41	13.27	74.35
G14	0.30	2.472	5.26	14.89	64.68
G15	0.25	2.421	7.44	16.8	55.72

**Table 11:** Variation in the void distribution fractal dimension (D_V_) with varying *D_f_*.

*D*_*f*_	Section Type	Section No.						Mean Value
		1	2	3	4	5	6	
		Void Fractal Dimension (D_V_)						
2.4	Transverse section	2.0595	2.0613	2.0628	2.0616	2.0629	2.0609	2.0615
2.5		2.0596	2.0579	2.0595	2.0591	2.06	2.0577	2.0590
2.6		2.0621	2.0621	2.0628	2.0633	2.0639	2.0637	2.0630
2.7		2.0615	2.062	2.0623	2.0607	2.0633	2.0613	2.0619
2.8		2.0639	2.0616	2.0603	2.0613	2.0627	2.0623	2.0620
2.4	Vertical section	2.0592	2.0628	2.0617	2.0617	2.0617	2.0547	2.0603
2.5		2.0658	2.0638	2.0616	2.0625	2.0674	2.0688	2.0650
2.6		2.063	2.0644	2.0606	2.0625	2.0687	2.0568	2.0627
2.7		2.0657	2.0638	2.0598	2.0588	2.0639	2.0642	2.0627
2.8		2.0637	2.0606	2.0628	2.0622	2.0639	2.0596	2.0621

**Table 12:** Factors and levels for the orthogonal experiment.

Factors	Level 1	Level 2	Level 3	Level 4	Level 5
*D_c_*	2.1	2.3	2.5	2.7	2.9
*D_f_*	2.4	2.5	2.6	2.7	2.8
k	0.25	0.3	0.35	0.4	0.45
P_b_	0.038	0.042	0.046	0.05	0.054
Compaction cycles	35	50	75	95	110

**Table 13:** Summary of mineral aggregate gradations synthesized based on *D_c_*, *D_f_*, and k.

No.	Gradation Parameters	Percent Passing (%)									
		16	13.2	9.5	4.75	2.36	1.18	0.6	0.3	0.15	0.075
1	(2.1, 2.4; 0.45)	100	95	73.7	45	29.6	19.5	13	8.6	5.7	3.7
2	(2.1, 2.5; 0.40)	100	95	71.6	40	28.2	19.9	14.2	10.1	7.1	5
3	(2.1, 2.6; 0.35)	100	95	69.4	35	26.5	20.1	15.3	11.6	8.8	6.7
4	(2.1, 2.7; 0.30)	100	95	67.3	30	24.3	19.8	16.1	13.1	11	8.6
5	(2.1, 2.8; 0.25)	100	95	65.2	25	21.7	18.9	16.5	14.4	13	10.9
6	(2.3, 2.4; 0.4)	100	95	72.9	40	26.3	17.3	11.6	7.6	5	3.3
7	(2.3, 2.5; 0.35)	100	95	70.9	35	24.7	17.4	12.4	8.8	6.2	4.4
8	(2.3, 2.6; 0.3)	100	95	68.8	30	22.7	17.2	13.1	9.9	7.5	5.7
9	(2.3, 2.7; 0.25)	100	95	66.8	25	20.3	16.5	13.4	10.9	8.9	7.2
10	(2.3, 2.8; 0.45)	100	95	74.9	45	39.1	34.1	29.7	25.9	23	19.6
11	(2.5, 2.4; 0.35)	100	95	72.3	35	23	15.2	10.1	6.7	4.4	2.9
12	(2.5, 2.5; 0.3)	100	95	70.4	30	21.1	15	10.7	7.5	5.3	3.8
13	(2.5, 2.6; 0.25)	100	95	68.5	25	18.9	14.3	10.9	8.3	6.3	4.8
14	(2.5, 2.7; 0.45)	100	95	76.1	45	36.5	29.6	24.2	19.7	16	13
15	(2.5, 2.8; 0.4)	100	95	74.2	40	34.8	30.3	26.4	23	20	17.4
16	(2.7, 2.4; 0.3)	100	95	71.9	30	19.7	13	8.7	5.7	3.8	2.5
17	(2.7, 2.5; 0.25)	100	95	70.1	25	17.6	12.5	8.9	6.3	4.4	3.1
18	(2.7, 2.6; 0.45)	100	95	77.2	45	34	25.8	19.7	14.9	11	8.6
19	(2.7, 2.7; 0.40)	100	95	75.4	40	32.4	26.3	21.5	17.5	14	11.5
20	(2.7, 2.8; 0.35)	100	95	73.7	35	30.4	26.5	23.1	20.1	18	15.3
21	(2.9, 2.4; 0.25)	100	95	71.7	25	16.4	10.8	7.2	4.8	3.1	2.1
22	(2.9, 2.5; 0.45)	100	95	78.4	45	31.7	22.4	16	11.3	8	5.7
23	(2.9, 2.6; 0.4)	100	95	76.7	40	30.2	22.9	17.5	13.3	10	7.6
24	(2.9, 2.7; 0.35)	100	95	75	35	28.4	23	18.8	15.3	12	10.1
25	(2.9, 2.8; 0.30)	100	95	73.4	30	26.1	22.7	19.8	17.3	15	13.1

**Table 14:** Orthogonal experimental scheme and test results of volumetric parameters.

No.	Factors and Levels					Test Indices			
	*D_c_*	*D_f_*	k	P_a_	Compaction Blows	Bulk Density	VV	VMA	VFA
1	2.1	2.4	0.45	0.38	35	2.371	9.9	16.77	40.91
2	2.1	2.5	0.4	0.42	50	2.451	6.6	14.48	54.64
3	2.1	2.6	0.35	0.46	75	2.515	3.9	12.81	69.9
4	2.1	2.7	0.3	0.5	95	2.524	3.2	13.06	75.29
5	2.1	2.8	0.25	0.54	110	2.5	3.9	14.43	73.3
6	2.3	2.4	0.4	0.46	95	2.458	5.7	14.58	60.6
7	2.3	2.5	0.35	0.5	110	2.492	4.2	13.94	70.17
8	2.3	2.6	0.3	0.54	35	2.444	5.7	16.12	64.58
9	2.3	2.7	0.25	0.38	50	2.422	9.1	15.82	42.24
10	2.3	2.8	0.45	0.42	75	2.505	4.5	12.69	64.45
11	2.5	2.4	0.35	0.54	50	2.399	7.2	17.5	58.78
12	2.5	2.5	0.3	0.38	75	2.398	9.8	16.45	40.61
13	2.5	2.6	0.25	0.42	95	2.406	9.1	16.68	45.16
14	2.5	2.7	0.45	0.46	110	2.543	2.4	11.61	79.6
15	2.5	2.8	0.4	0.5	35	2.532	2.6	12.62	79.46
16	2.7	2.4	0.3	0.42	110	2.39	9.5	17.07	44.21
17	2.7	2.5	0.25	0.46	35	2.336	11.3	19.44	42.04
18	2.7	2.6	0.45	0.5	50	2.518	2.7	12.8	78.67
19	2.7	2.7	0.4	0.54	75	2.527	2.2	13.08	83.53
20	2.7	2.8	0.35	0.38	95	2.514	5.4	12.44	56.94
21	2.9	2.4	0.25	0.5	75	2.353	10.1	19.21	47.33
22	2.9	2.5	0.45	0.54	95	2.502	2.7	13.65	79.92
23	2.9	2.6	0.4	0.38	110	2.539	4	11.29	64.15
24	2.9	2.7	0.35	0.42	35	2.498	5.3	13.27	60.01
25	2.9	2.8	0.3	0.46	50	2.512	4.5	13.4	66.1

**Table 15:** Grey relational analysis results of the five investigated factors.

Rank	Factors	GRA with VV	Factors	GRA with VMA	Factors	GRA with VFA
1	Compaction cycles	0.627	Compaction cycles	0.62	Compaction cycles	0.743
2	P_b_	0.565	*D_c_*	0.604	P_b_	0.707
3	*D_c_*	0.565	P_b_	0.574	*D_c_*	0.677
4	k	0.546	k	0.545	k	0.647
5	*D_f_*	0.534	*D_f_*	0.542	*D_f_*	0.586

**Table 16:** Volumetric and interfacial indicators of optimized and conventional AC-13 mixtures (P_b_ = 5.0%).

Aggregate Gradation Type	Bulk Density (g/cm^3^)	VV (%)	VMA (%)	VFA (%)	$\mathrm{Total}\;\mathrm{SA}\;(m^{2}/kg)$	AFT (μm)
OG	2.480	4.35	14.53	66.66	3.40	15.03
CG	2.509	3.72	13.53	72.47	5.93	8.61

**Table 17:** Experimental results of pavement performance validation for asphalt mixtures.

Mixture Type	Tensile Strength Ratio (%)	Dynamic Stability (Times/mm)	Flexural-Tensile Strength (MPa)	Flexural-Tensile Strain (με)
OG	87.6	2617	9.96	2.658
CG	86.5	1230	10.02	2.712

## Data Availability

The original contributions presented in this study are included in the article. Further inquiries can be directed to the corresponding author.
